# Membrane Lipid Composition Influences the Hydration of Proton Half-Channels in F_o_F_1_-ATP Synthase

**DOI:** 10.3390/life13091816

**Published:** 2023-08-28

**Authors:** Leonid A. Ivontsin, Elena V. Mashkovtseva, Yaroslav R. Nartsissov

**Affiliations:** 1 Institute of Cytochemistry and Molecular Pharmacology, 24/14 6th Radialnaya Str., Moscow 115404, Russia; elenamash@gmail.com (E.V.M.); yn_brg@icmph.org (Y.R.N.); 2 Biomedical Research Group, BiDiPharma GmbH, 5 Bültbek, 22962 Siek, Germany

**Keywords:** membrane proteins, F_o_F_1_-ATP synthase, cardiolipin, proton transport, molecular dynamics

## Abstract

The membrane lipid composition plays an important role in the regulation of membrane protein activity. To probe its influence on proton half-channels’ structure in F_o_F_1_-ATP synthase, we performed molecular dynamics simulations with the bacterial protein complex (PDB ID: 6VWK) embedded in three types of membranes: a model POPC, a lipid bilayer containing 25% (in vivo), and 75% (bacterial stress) of cardiolipin (CL). The structure proved to be stable regardless of the lipid composition. The presence of CL increased the hydration of half-channels. The merging of two water cavities at the inlet half-channel entrance and a long continuous chain of water molecules directly to *c*Asp61 from the periplasm were observed. Minor conformational changes in half-channels with the addition of CL caused extremely rare direct transitions between *a*Glu219-*a*Asp119, *a*Glu219-*a*His245, and *a*Gln252-*c*Asp61. Deeper penetration of water molecules (W1–W3) also increased the proton transport continuity. Stable spatial positions of significant amino acid (AA) residue *a*Asn214 were found under all simulation conditions indicate a prevailing influence of AA-AA or AA-W interactions on the side-chain dynamics. These results allowed us to put forward a model of the proton movement in ATP synthases under conditions close to in vivo and to evaluate the importance of membrane composition in simulations.

## 1. Introduction

Living cells need to constantly accumulate and transfer energy to carry out their activities. For this, they produce molecules called adenosine triphosphate (ATP) containing high-energy bonds [1]. Most cellular ATP is synthesized by the F_o_F_1_-ATP synthase protein complex found in the bacterial cytoplasmic membrane, chloroplast thylakoids, and the inner mitochondrial membrane [2]. The enzyme reversibly couples the proton gradient across energy-transducing membranes to the synthesis of ATP from ADP and P_i_ [3]. It consists of two rotary motors: a hydrophilic F_1_ that has catalytic sites for ATP synthesis/hydrolysis, and a membrane-embedded F_o_ that mediates proton transfer [4,5]. The operation of the F_1_ motor has been studied and described fairly well, unlike the transmembrane proton pump F_o_.

Recently, many structural models of F_o_-ATP synthase with near-atomic resolution have been obtained using cryoelectron microscopy. This elucidates on many details of how the membrane part of the enzyme functions [6,7,8]. The two components of F_o_ that are directly involved in the transport process are a stator *a*-subunit containing two non-aligned half-channels through which protons can pass from the periplasm/cytoplasm and a rotating oligomer of *c*-subunits (*c*-ring) with key amino acid residues (aspartate or glutamate) that accept and donate protons [9]. However, the exact pathway of proton transport across the membrane at atomic resolution remains elusive, as does the mechanism of the close coupling between the proton transport and *c*-ring motion.

Despite slight differences in the subunit structure in various organisms, the catalytic cycle of the protein complex is highly conserved. In particular, during ATP synthesis in *Escherichia coli*, protons moving from the periplasm along the inlet half-channel bind to the conserved *c*Asp61 near the middle of the *c*-subunit and are released to the cytoplasm when the same *c*-subunit reaches the outlet half-channel after turning almost 360°. In the case of hydrolysis, the process goes in the opposite direction [9,10]. These processes are observed in all cases when any sourced ATP synthase is examined. Therefore, the investigation of a single enzyme from *E. coli* is worthwhile and may lead to a keen insight into the functioning of ATP synthase in all species.

A large amount of evidence has been obtained for particular interactions between lipids and membrane proteins, as well as lipids’ specific type influence on enzyme functioning by X-ray crystallography, electron microscopy, and molecular dynamics (MD) simulations [11]. Typically, model membranes, such as 1-palmitoyl-2-oleoyl-phosphocholine (POPC) or 1-palmitoyl-2-oleoyl-phosphatidylethanolamine (POPE), are used in protein simulations in lipid membranes. However, the specific anionic phospholipid, cardiolipin (CL), having four acyl chains and two phosphate groups, plays an important role in the regulation of oxidative phosphorylation (OxPhos) in both animals and microorganisms [12]. Bacterial cells have phosphatidylethanolamine (PE) as their most abundant zwitterionic lipid (~75%) and also contain 20–25% of negatively charged lipids in their cytoplasmic membranes, including phosphatidylglycerol (PG) and cardiolipin (CL) [13]. It is believed that the presence of PE and CL is a prerequisite for optimal activity of respiratory chain proteins, including complex I (NADH/quinone oxidoreductase), complex III (cytochrome bc_1_), complex IV (cytochrome c oxidase), and ATP synthase [14,15,16]. Although PE and CL are non-bilayer-forming lipids, experimental evidence has demonstrated that they are present in the lipid bilayers in vivo. Moreover, they locally can form curved structures under certain conditions and with considerable energy input [17].

It was shown that CL connects strongly to highly specific binding sites in complex III and complex IV with long residence times, and due to the lack of CL, enzyme activity was reduced [18,19]. CL-protein interactions are not limited to the OxPhos enzymes, as evidenced by a rather large list of other proteins whose work is specifically associated with CL: ADP/ATP carriers, osmosensory transporters, and translocases [20,21,22]. Moreover, it was shown that CL affects the conformational changes and rigidity of proteins [23] and folding [24] promotes the formation of dimeric rows of ATP synthase [25]. In addition, an increased content of CL additionally ensures the survival of bacteria in adverse conditions [21]. Although the presence of CL is not essential, it does have an impact on the ATP synthase activity [26]. Previous studies have confirmed the existence of highly specific transient interactions with the rotating *c*-ring, which result in the stabilization and lubrication of the rotor [27,28]. Additionally, there is an assumption that CL interacts with the enzyme at the inlet and the outlet of the transmembrane half-channels functioning as a proton trap and localizing the flow of protons in the near-membrane layer [16,29]. However, the effect of cardiolipins on proton transport is not well understood.

Adaptation to bacterial stress depends on the successful development of the appropriate cellular response, which includes cell membrane remodeling, changes in gene expression, or metabolic adjustments [30]. All these processes depend on the cells’ ability to provide energy that is usually produced by F_o_F_1_-ATP synthase. For *E. coli*, an increase in the cardiolipin content is an important mechanism of adaptation to the conditions of stress and energy metabolism disorder, however, the upper limit of CL concentration is still unknown [21]. It is believed that such modifications of the cytoplasmic membrane maintain the stability of the lipid bilayer configuration [31]. Experimental research has successfully obtained mutant *E. coli* strains with a high content of CL, showing viability under specific circumstances. The other results suggest that a respiratory chain and F_o_F_1_-ATP synthase still work, increasing survival in extremely adverse conditions [30,32]. However, the structural and functional stability of the membrane part of ATP synthase, in this case, is still not clear. Nevertheless, the content variation of main phospholipids increases oxidative stress and it has a profound effect on energy metabolism [30]. Thus, molecular dynamics simulation of the protein–membrane complex with a high level of CL will provide valuable insights into the unresolved questions.

Previously, we have studied the structure of proton half-channels in the model POPC membrane. Molecular dynamics simulation of the F_o_ factor of *E. coli* ATP synthase embedded in the lipid bilayer and water environment have offered important insights into the proton transport pathway, since the static information from cryo-EM structures cannot on its own explain how and where protons pass through F_o_. The structural dynamics of significant polar amino acids and protein hydration have been investigated. Conservative localizations of structural water molecule clusters critical for proton transport have been established [33]. However, the study of membrane lipid composition’s influence on the structure of proton half-channels is especially interesting. In the present study, the models of both in vivo membrane containing 25% CL, and lipid bilayer with 75% CL that corresponds to the conditions of bacterial stress were considered with comparison to the POPC membrane model. We varied the levels of cardiolipins, which play a significant role in energy transducing processes maintaining the structure and functional activity of the ATP synthase. The obtained results clearly indicate that the presence of CL apparently increased the hydration of half-channels. The identified regions of possible proton pathways across the membrane allow us to put forward a microscopic model of the mechanism of proton movement in ATP synthases under conditions approximate to in vivo, as well as evaluate the importance of membrane composition in simulations.

## 2. Materials and Methods

**Figure 1:**
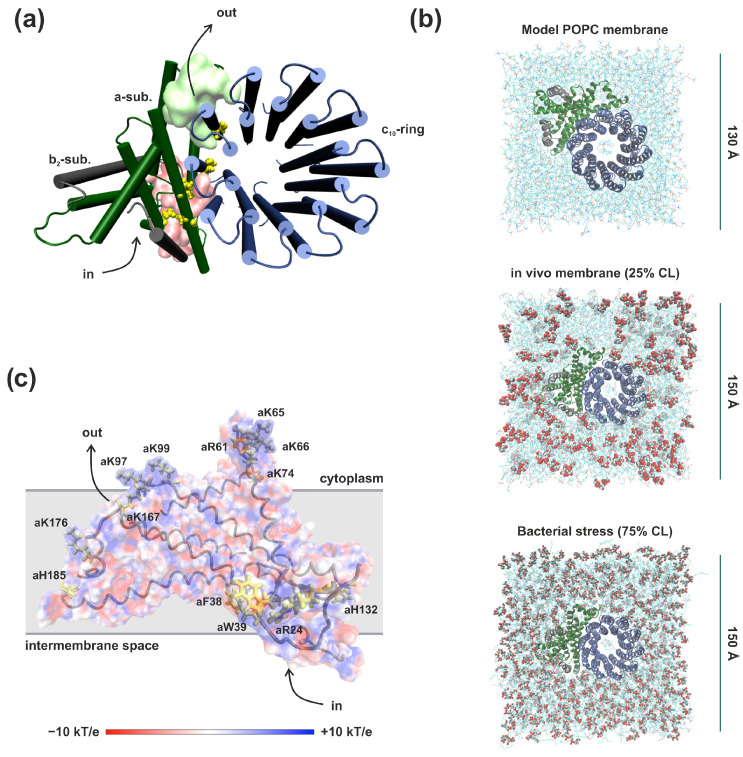
Structural characteristics of molecular model systems. (**a**) Simulation setup of the *E. coli* F_o_F_1_-ATP synthase membrane part. Hereafter, F_o_ subunits are shown: *a*-subunit (green), *b*-subunit (black), and *c_10_*-ring (blue). Polar amino acid residues presumably involved in proton transport are labeled as yellow balls. The surface shows the areas of the input (pink) and output (lime) half-channels. The protein is shown from the top view (from the cytoplasm). The arrows indicate the intended direction of proton movement during ATP synthesis. (**b**) The three types of membranes studied were: a model POPC membrane, an in vivo *E. coli* membrane, and a simulated bacterial stress. The polar head groups of cardiolipins are labeled as VDW and licorice. (**c**) Electrostatic potential map of ATP synthase *a*-subunit (side view). The color scale ranges from −10 kT/e to +10 kT/e. Amino acids interacting with cardiolipins are labeled.

Molecular dynamics simulations were used to study the membrane lipid composition’s effect on the proton half-channels’ structure of F_o_F_1_-ATP synthase in up to microsecond timescales. The molecular model system was a membrane part of the enzyme embedded in a lipid bilayer. The protein portion was constructed from the Cryo-EM crystal structure of *E. coli* (PDB ID: 6VWK) [8] and comprised *a*-subunit, ring of ten *c*-subunits, and two truncated *b*-subunits (residues 4 to 35) (Figure 1a). Titratable residues were modeled based on protonation states obtained by the H++ webserver [34]. In *a*-subunit, all significant polar amino acids (including *a*Asp119, *a*Ser144, *a*Arg210, and *a*Glu219) were considered to be ionized and *a*His245 was modeled as neutral with the proton on NE. The key *c*Asp61 of all *c*-subunits was in the protonated state, except for aspartate facing the lumen of the inlet half-channel.

The protein structure described above was embedded in three types of membranes: a model POPC membrane, an in vivo *E. coli* membrane (CL:PE ratio of 1:3), and a simulated bacterial stress (CL:PE ratio of 3:1) (Figure 1b). In the model membrane, the protein was embedded into the 1-palmitoyl-2-oleoyl-phosphatidylcholine (POPC) bilayer with the Membrane Builder module of CHARMM-GUI [35]. For in vivo simulation, three membranes with a CL:PE ratio of 1:3 (25% CL) in which lipids were placed randomly in the initial configurations were constructed. To mimic bacterial membrane multiple types of acyl chains (1,2-dipalmitoyl-1′-palmitoil-2′-cis-9,10-methylenehexadecanoyl-cardiolipin (PMCL2), 1,2-1′,2′-tetrahexadecenoyl-cardiolipin (TYCL2), 1,2-dipalmitoleic-phosphatidylethanolamine (DYPE), 1,2-dipalmitoyl-phosphatidylethanolamine (DPPE), and 1-palmytoil-2-cis-9,10-methylenehexadecanoyl-phosphatidylethanolamine (PMPE)) have been considered [35]. The three membranes simulating bacterial stress were randomly constructed with the same types of lipids and a CL:PE ratio of 3:1 (75% CL). The stability of the lipid bilayer containing 75% CL was shown in [35] using the MD simulation. In all obtained models one PC or PE lipid was inserted inside the c-ring in each leaflet of the membrane. The systems were solvated with TIP3P water molecules [36] and also neutralized with 150 mM KCl. The full model system included ca. 130,000 atoms and the total size was estimated as 130 Å × 130 Å × 90 Å for POPC or ca. 220,000 atoms and 150 Å × 150 Å × 90 Å for CL.

The MD simulations were conducted using NAMD software version 2.14 [37], in conjunction with the CHARMM36 force field [38,39]. A constant temperature and pressure (NPT ensemble) were maintained at 310 K and 1 bar, respectively, through the application of a Langevin thermostat and an anisotropic Langevin piston barostat. Van der Waals interactions were calculated with a 12 Å cutoff, incorporating a switch after 10 Å. The particle mesh Ewald (PME) method, with a 12 Å cutoff, was employed to compute long-range electrostatic interactions. The time step was 2 fs, and the coordinates were recorded every 2 ps. Initially, the model systems underwent energy minimization, followed by a 15 ns equilibrium period within the NPT ensemble, with protein atoms harmonically restrained by a force constant of 2 kcal mol-1 Å-2. Subsequently, MD simulations proceeded without restrictions. Three independent simulations (run1–3) of a model POPC membrane were conducted for 150 ns each, with the only difference being random initial velocities. Systems involving in vivo membranes (run4–6) and simulated bacterial stress (run7–9) were simulated for 300 ns each. Visualization and analysis of the resulting trajectories were performed using the VMD program [40] and custom Tcl scripts.

We studied the proton half-channels’ structure of ATP synthase, of particular interest was the determination of the possible proton trajectory. For these purposes the mutual arrangement of amino acids and water which can have a significant effect on proton transport were considered. It is believed that the polar groups of titratable amino acids, as well as the oxygen atoms of water molecules, can influence the proton motion, forming short-lived bound states with H^+^ [41]. Moreover, the amino acid residues’ side chains of asparagine and glutamine can function as acceptors/donors in the proton transfer pathways due to the formation of the enol species [42]. During the analyzing of MD trajectories, we detected the atoms of significant amino acids and water in half-channels possibly involved in the formation of the H-bond as located at a distance of up to 3 Å (approximate length of the hydrogen bond). We believe that there is a possibility of direct proton transfer between these atoms and they could be involved in the proton transfer chain, although the H^+^ transfer itself was not simulated.

## 3. Results

### 3.1. Proton Transport Pathway in the F_o_ Factor of E. coli ATP Synthase Embedded in Model POPC Bilayer

**Figure 2:**
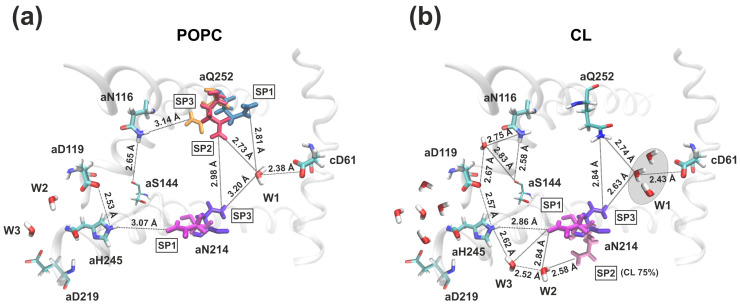
Scheme of the possible proton translocation pathway in the inlet half-channel of F_o_F_1_-ATP synthase in model POPC membrane (**a**), in vivo membrane containing 25% CL and 75% CL (bacterial stress conditions) (**b**). Significant charged amino acid residues and water molecules are labeled as licorice. Stable spatial positions SP1, SP2, and SP3 of the side chains *a*Asn214 are shown in magenta, mauve, and violet, SP1, SP2, and SP3 of *a*Gln252–blue, pink, and orange, respectively. The distances indicate the minimum observed values along the MD trajectories.

**Figure 3:**
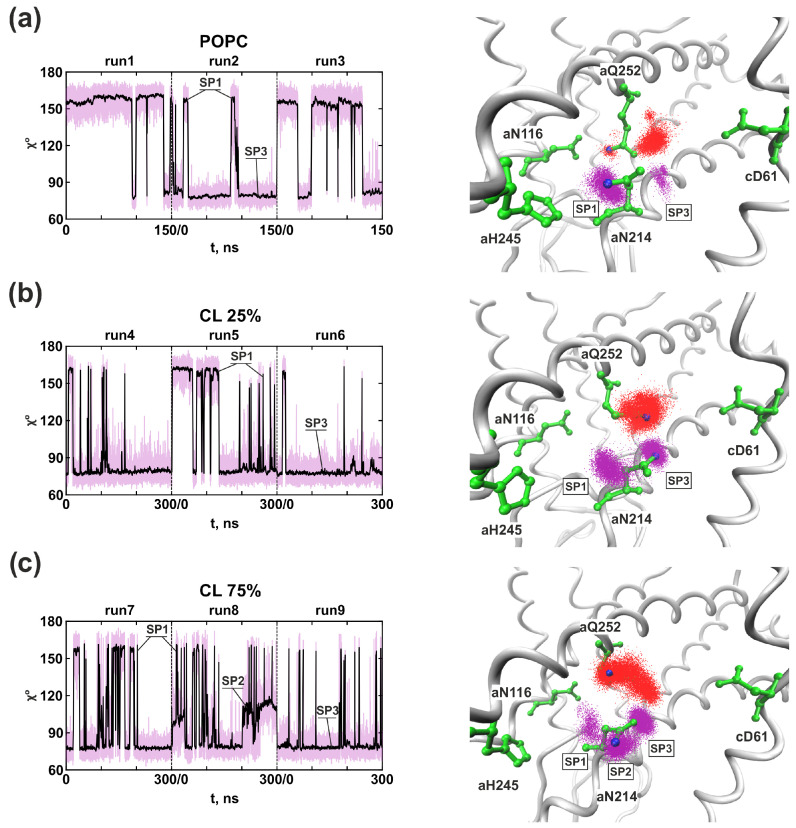
Stable spatial positions (SPs) of the side chains of *a*Asn214 in model POPC membrane (**a**), in vivo membrane containing 25% CL (**b**) and 75% CL (bacterial stress conditions) (**c**). On the left, is the time propagation of the angle χ values between the side chain and the axis of the alpha helix in three independent runs. On the right, are schemes of the spatial positions of significant amino acid residues and water molecules involved in proton transfer (labeled as licorice) with probability maps of the *a*Asn214 ND2 atom location (magenta dots) and *a*Gln252 NE2 atom (red dots). Significant amino acid residues involved in proton transfer are labeled as green licorice. With the adjunction of CL, *a*Asn214 retains distinct spatial positions (SP1 and SP3), while the SPs of *a*Gln252 were mixed (**b**,**c**).

In model POPC membrane, it was discovered that the inlet half-channel had a complex structure with two aqueous lacunae, through which the proton could penetrate into the half-channel and reach the significant amino acid residue *a*Glu219 of the *a*-subunit. The further proton path from *a*Glu219 to the key *c*Asp61 of the *c*-subunit passed through the chain of amino acid residues and structural water molecules: *a*Glu219–W3–W2–*a*Asp119–*a*His245–*a*Asn214–*a*Gln252–W1–*c*Asp61 (Figure 2a). The distances between *a*Glu219–*a*Asp119 and *a*Gln252–*c*Asp61 exceeded the threshold of 3 Å, so direct proton transfer between them was impossible. However, the localization of three structural water molecules (W1–W3) which were necessary for the proton transport chain continuity were determined around them. In addition, the side chains of some amino acid residues had a very high mobility between their stable spatial positions (SPs). Stable spatial positions of the *a*Asn214 and *a*Gln252 *a*-subunit were found and named SP1, SP2, and SP3. In particular, it was established that *a*Asn214 in SP1 was oriented to *a*His245, and in SP3 to *c*Asp61, and change-over between them resembled an operation like a switcher between elements of an electric chain (Figure 2a and Figure 3a). Thus, the proton transfer chain is always unclosed, and shifting between positions SP1 and SP3 of *a*Asn214 determines the time of proton transport. At the same time, the outlet half-channel was a water cavity and contained a large number of hydrophilic amino acid residues, that, together with water molecules, formed a vast network of hydrogen bonds, along which the proton could move from *c*Asp61 to the cytoplasm.

Thus, the localization of proton half-channels as well as a possible proton transport chain were identified in bacterial ATP synthase (PDB ID: 6VWK) embedded in the model POPC membrane. Nevertheless, the insufficient probability of direct transitions between significant amino acid residues in the inlet half-channel, in combination with low protein hydration, indicated a high risk of proton transport instability. The continuity of the transfer chain was ensured by the need for the simultaneous occurrence of rare events (direct transfer between amino acids, SP, and the presence of water molecules in certain regions). It is possible that the variability of the membrane lipid composition, including the presence of charged phospholipids (CLs), affects the internal state of the half-channels through the stabilization of preferred amino acid positions and increased hydration.

### 3.2. Membrane Lipid Composition Influence on the Structure and Hydration of the Proton Half-Channels

To probe the membrane lipid composition’s effect on the structure and hydration of the membrane part of F_o_F_1_-ATP synthase, we performed molecular dynamics simulations on microsecond time scales with the bacterial protein complex embedded in a mimic in vivo lipid bilayer containing 25% CL and a lipid bilayer with 75% CL that simulated bacterial stress (Figure 1b). In order to evaluate the consistency of the simulated system, three runs were conducted for each type (run4–6 for 25% CL and run7–9 for 75% CL). For each run, the initial configurations of lipids were randomly generated. In the resulting structures, the lipids were neatly packed around the protein and the root mean square deviations of all C_α_ atoms (RMSD) were less than 3 Å, indicating that the overall stability of the enzyme was not significantly affected by adjunction CL to the system.

To detect regions of interaction with cardiolipins on the surface of the *a*-subunit, binding sites were determined as a region of the protein surface located at a distance of up to 3 Å from the phosphatidic acid moieties in CL. Despite the various initial positions of CL, MD simulations showed approximately ten CL binding sites in the *a*-subunit indistinguishable in all runs. Although the average CL retention time was slightly increased in membranes simulating bacterial stress, CL-protein interactions were transient, in contrast to complex III or IV of the respiratory chain, where cardiolipin molecules strongly modulate the protein activity. CL binding is mainly induced by positively charged amino acids (Lys, Arg, His) or aromatic residues (Phe, Trp) located at the lipid–protein interface (Figure 1c). Moreover, some of these binding sites are located in close proximity to the transmembrane helices that form proton half-channels, in particular, near the entrance to the inlet half-channel.

**Figure 4:**
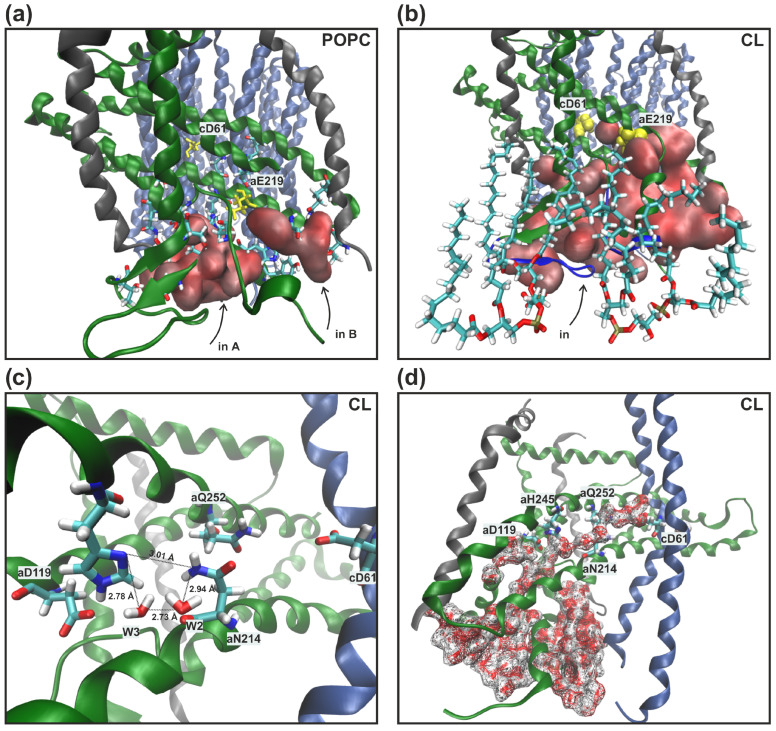
Water accessibility of the inlet half-channel obtained from simulations in the model POPC membrane [33] (**a**) and an in vivo membrane containing 25% CL (**b**). The separate (**a**) and aggregate (**b**) aqueous lacunae are labelled as a red surface, cardiolipins are drawn as licorice. Significant *a*Glu219 and *c*Asp61 are shown in yellow. *a*-subunit loops (amino acids 27–37 and 133–140) with high mobility in the membrane with CL are highlighted in blue. (**c**) Detailed view of *a*Asn214 and *a*His245 (view from the *b*-subunit) in the membrane with CL. Polar amino acids residues and water molecules involved in proton transfer are labeled as licorice. Deeper penetration of water molecules W2 and W3 coordinate the proton transition between *a*His245 and *a*Asn214. The distances indicate the minimum observed values in simulations. (**d**) A continuous water molecule chain, through which the proton could reach the *c*Asp61 directly from the aqueous lacuna entrance, is represented by the wireframe surface.

**Figure 5:**
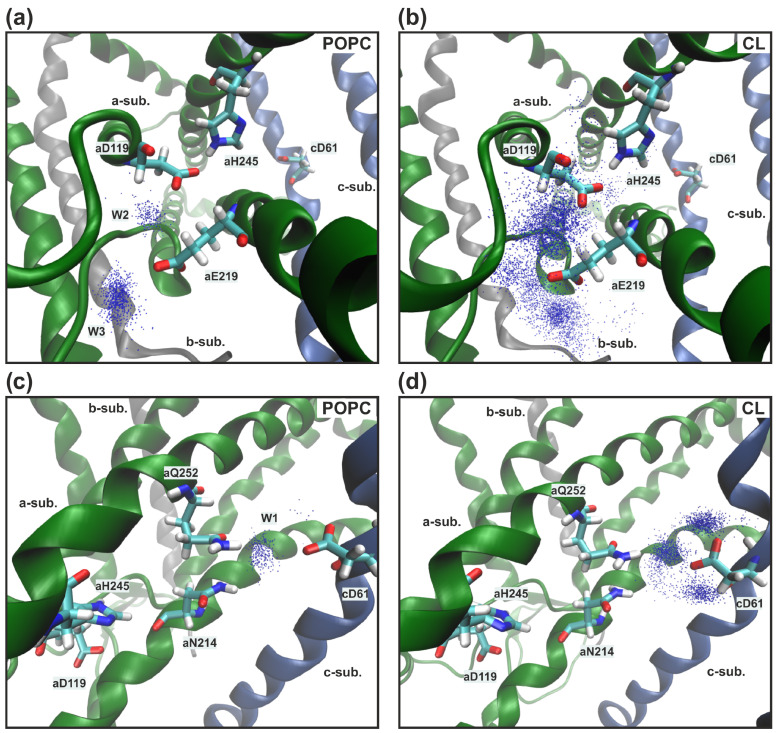
(**a**,**b**) Probability maps of the water molecule’s location at a distance of less than 3 Å from *a*Asp119, *a*Glu219, and *a*His245 (blue dots) across MD simulations in the model POPC membrane (**a**) and an in vivo membrane with CL (**b**). In the POPC membrane two water molecules (W2 and W3) coordinate the transition between *a*Glu219 and *a*Asp119 (**a**). There is an increased hydration of the region in the presence of CL (**b**). (**c**,**d**) Conserved chain of significant amino acid residues (*a*Asp119, *a*Asn214, *a*His245, *a*Gln252, and *c*Asp61) in the inlet half-channel are labeled as licorice. View from the *b*-subunit. Probability maps of the water molecule’s location at a distance of less than 3 Å from *a*Asn214, *a*Gln252 and *c*Asp61 (blue dots) across MD simulations in model POPC membrane (**c**) and in vivo membrane with CL (**d**). The presence of CL contributes to the penetration of more water molecules into this region, forming W1 as a cluster consisting of 2–3 structural water molecules.

In all MD trajectories two or three cardiolipins came into contact with polar amino acid residues lining aqueous lacuna, through which the proton presumably entered the inlet half-channel. *a*Arg24 and *a*His132 were CL binding sites, and their transient interactions led to high mobility of the adjacent *a*-subunit loops (amino acids 27–37 and 133–140) that form the walls of water cavities. As a result, we observed the penetration of more water molecules into the lacuna and one aggregate entrance, through which the proton could reach the essential *a*Glu219 and not two separate ones as in the model POPC membrane (Figure 4a compared with Figure 4b). By overlaying a large number of separate MD coordinate frames, a residence probability map of stably located water molecules can be obtained (Figure 5a,b, blue dots). In the presence of CL, the region near *a*Glu219, *a*Asp119, and *a*His245 has significantly increased hydration (Figure 5a compared with Figure 5b). Water molecules penetrated into the inlet half-channel up to these amino acids, forming very branched chains of hydrogen bonds, through which the proton could easily reach them from the periplasm, for example, by the Grotthuss mechanism. Remarkably, in the model POPC membrane we observed two structural water molecules (W2 and W3) (Figure 5a) which continued the proton transfer chain [33], since there was no direct transfer from *a*Glu219 to *a*Asp119/*a*His245. Whereas, in in vivo membranes and simulated bacterial stress with CL, W2 and W3 still existed, but a possible direct transfer was observed. However, the probability of such a transition was extremely low since the contact (the distance was less than 3 Å) between amino acids was transient. Additionally, *a*Asp119 and *a*His245 were in direct contact for more than 90% of the simulation time both with and without CL.

**Figure 6:**
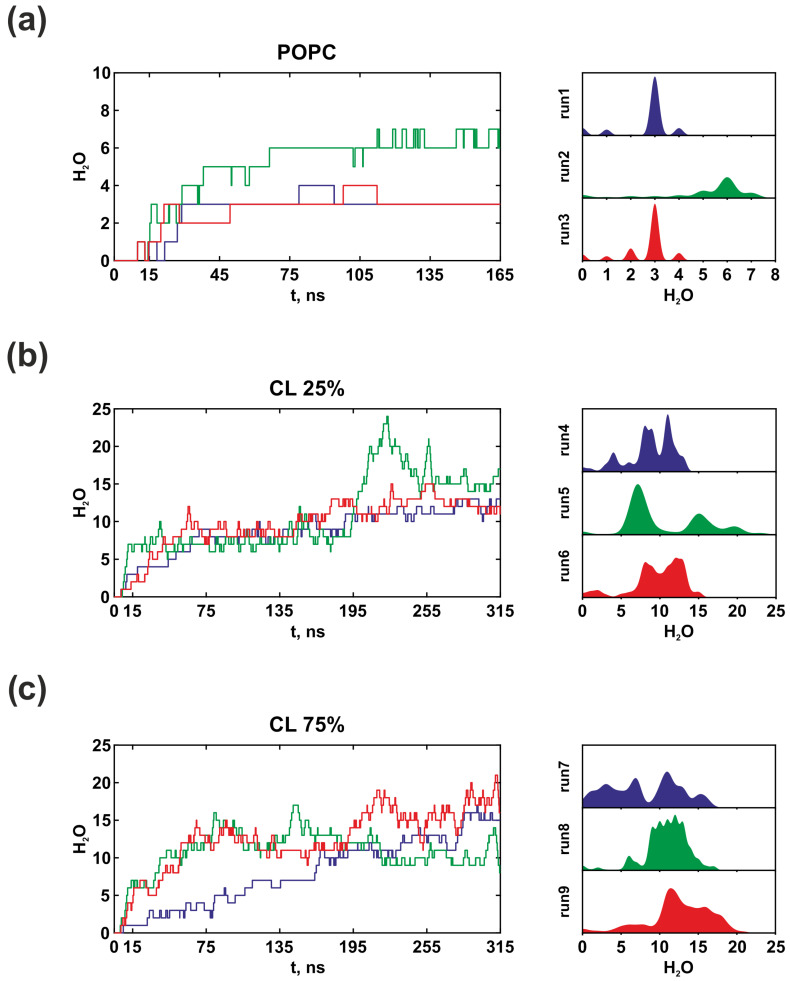
Hydration dynamics of the inlet half-channel during MD simulations in the model POPC membrane (**a**), in vivo membrane containing 25% CL (**b**) and 75% CL (bacterial stress conditions) (**c**). On the left, is the time propagation of the water molecules’ number located at a distance of 5 Å from significant amino acid residues (*a*Asn116, *a*Asp119, *a*Ser144, *a*Asn214, *a*His245, and *a*Gln252) inside the inlet half-channel in three runs (blue, green and red lines). On the right, are probability density estimations. Each color of the graph on the right corresponds to the color of the line on the left. The presence of CL obviously increased the hydration of the inlet half-channel.

Further proton transfer from *a*His245 was carried out to *a*Asn214 (Figure 2). However, due to the high mobility of the *a*Asn214 side chains, the distance between them varied from 3 Å to 12 Å in all types of membranes. In the stable position SP1, when *a*Asn214 was oriented to *a*His245, the average distance between them was 4.41 Å. Moreover, it rarely took a value less than 3 Å, thereby it made it difficult for protons to move in this region. In membranes with CL, increased hydration (Figure 6) of the antecedent proton motion region has led to deeper penetration of water molecules W2 and W3 inside the protein, now responsible for the coordination of the proton transfer from *a*His245 to *a*Asn214 (Figure 4c).

The last step in the proton transport chain in the inlet half-channel is the transition to *c*Asp61, that can be carried out from *a*Asn214 or *a*Gln252, however, their side chains had a high mobility. To describe their dynamics, the root mean square fluctuation (RMSF) of the amino acid side chain and the angle χ between the side chain and the axis of the alpha helix were calculated (Figure 3). Amino acid side chains’ stable spatial positions were determined as positions having average fluctuation amplitudes on par with the magnitude of atoms’ thermal fluctuations [33]. Regardless of the CL presence in the membrane, stable spatial positions of *a*Asn214 were observed. If there was a 25% CL content in the in vivo membrane, *a*Asn214 had two stable positions (SP1 and SP3). In position SP1, it was directed to *a*His245, while it was more often observed to be oriented towards *c*Asp61 in position SP3. Under bacterial stress with 75% CL during the MD simulation in the run8, we found another stable spatial position of *a*Asn214, called SP2 (Figure 3c). In SP2, *a*Asn214 was in an intermediate position between *a*His245 and *c*Asp61, and it was oriented to the structural water molecule W2, which also penetrated deeper into the half-channel. The appearance of this spatial position was associated with a significant hydration increase (W2) near *a*His245/*a*Asn214, and lead to a magnification in the probability of proton transfer via this region. In the presence of CL, *a*Gln252 did not have distinct spatial positions. A direct transition between *a*Gln252 and *a*Asn116 (considered a dead end in the POPC membrane) was impossible. The overall structural mobility of the side chains of amino acid residues in the region near the key *c*Asp61 most likely does not directly depend on the CL content in the membrane. However, it is determined exclusively by local interactions between amino acids and water molecules.

In the model POPC membrane, proton transfer from *a*Asn214 and *a*Gln252 was carried out to *c*Asp61 only via the structural water molecule W1 that we discovered in all simulations [33] (Figure 5c). In the presence of CL, instead of the single favorable position of the structural molecule of water W1, a greater conformational freedom of water around *c*Asp61 was found. Therefore, the indication W1 now describes the several molecules due to the increased hydration in membranes containing CL. The most probable localizations of water molecules were determined (Figure 5d, blue dots), which were located at a distance of less than 3 Å from *c*Asp61. It can currently be easily protonated through the cluster W1 consisting of 2–3 structural water molecules. In addition, we detected the formation of a long continuous chain of well-ordered water molecules, through which the proton could reach the key *c*Asp61 from the aqueous lacuna entrance directly. Nevertheless, this chain was constantly broken in the region of significant amino acid residues *a*Asp119, *a*His245, and *a*Asn214, *a*Gln252, that confirms their importance in the proton transport process (Figure 4d).

Figure 6 illustrates that water molecules can quickly penetrate deep into the inlet half-channel, even during the equilibrium stage. The dynamics of hydration indicate that water remains in the half-channel for the entire simulation and does not leave in bulk. By conducting a thorough analysis, it was discovered that water molecules tend to localize in regions critical for proton transfer (W1-W3), highlighting their structural significance and necessity in the proton transport chain through F_o_F_1_-ATP synthase. Simultaneously, the presence of CL in the membrane promotes hydration, leading to enhanced stability in the continuity of proton transport chain, as well as an increase in the probability of protonation of the key *c*Asp61 under conditions close to in vivo.

Polar *a*Asn116 and *a*Ser144 were located outside of the “main H^+^ route”. In the model POPC membrane, no water molecules were found near them, and the proton could reach them in rare cases only through *a*Gln252 in position SP3. In the membrane with CL, we observed occasional cases of penetration of one or two water molecules via which the proton could pass from *a*Asp119 to *a*Asn116–*a*Ser144. Therefore, the importance of their participation in proton transfer is under discussion and these amino acids are still considered to be a dead end and they may represent a proton trap. It is not excluded that they do not participate in the proton transport process at all.

Regardless of the membrane type, the outlet half-channel was a cavity filled with water. *c*Asp61 was in contact with water molecules for more than 95% of the simulation time and could be deprotonated through them only, since the nearby polar amino acid residues were located at a distance exceeding 3 Å. At the same time, increased hydration of the cavity was observed in membranes with CL. On average, there were approximately 50–60 water molecules in the outlet half-channel (38–46 molecules in the model POPC membrane). Both water moleculesand polar amino acids residues could form a large network of hydrogen bonds through which the proton could easily move to the cytoplasm.

An increased content of CL did not significantly affect the overall stability of the membrane part of the protein complex, and it did not lead to undesirable conformational changes. The proton half-channels’ structure remained unchanged, except for the minor modification in the structural mobility of the *a*Asn214 side chain. Nevertheless, such a modification does not lead to a break in the proton trajectory, causing a disruption in the process of proton pumping through the membrane under bacterial stress.

## 4. Discussion and Conclusions

Proton transport through the F_o_ membrane factor of ATP synthase initiates the catalytic cycle of the enzyme. The energy of the transmembrane proton gradient induces rotation of the *c*-ring in F_o_, which carries energy to the catalytic domain in F_1_ to activate ADP phosphorylation [43]. Wherein, the exact trajectories of the proton movement are underexplored, in contrast to the ATP synthesis reaction itself.

Despite differences in subunits across species, the energy coupling mechanism is conserved, making it attractive to study bacterial ATP synthase as a simple example. In our previous work, we described the location and structure of both half-channels in *E. coli* ATP synthase embedded in the model POPC bilayer, as well as the possible network of polar amino acid residues that can contribute to the preferred proton pathway. In addition, the penetration of a large number of water molecules, which are also important for the biological functioning of the enzyme, has been established. We observed a large clustering of water molecules in the lacunae at the entrance and exit of the half-channels. Moreover, several structural water molecules (W1–W3) were found that had a high probability to be located in certain regions of the protein. They did not diffuse in bulk and were essential for the proton transfer chain to be completed.

Thus, MD simulations of the F_o_ fraction embedded in the model POPC membrane turned out to be an acceptable method for studying the proton half-channel’s structure. However, the insufficient probability of direct transitions between significant amino acid residues in the inlet half-channel, in combination with low protein hydration, indicated a high risk of proton transport instability. But ensuring the continuity of the proton transfer chain is a crucial moment in the functioning of living systems under various conditions. In this situation, it was especially important to determine the contribution of the membrane lipid composition or the structural characteristics of the protein. Therefore, we adapted our MD simulations to conditions close to in vivo.

Until recently, it was believed that membrane proteins float freely within biological membranes, but now highly specific interactions between lipids and membrane proteins have been determined with absolute certainty. In particular, it has been established that the anionic lipid cardiolipin has a significant effect on the processes of oxidative phosphorylation, the functioning of respiratory chain proteins, and ATP synthase. Usually, coarse-grained modeling is used to describe lipid–protein interactions, in which a reduction in the level of detail allows the study of biomolecular systems on much longer time scales. However, with such modeling, the details of specific interactions may be lost. Therefore, in order to realistically simulate and obtain adequate data, we performed atomistic molecular dynamics simulations on microsecond time scales with a bacterial protein complex (PDB ID: 6VWK) embedded in an in vivo membrane with a CL:PE ratio of 1:3 (25% CL), as well as lipid bilayer with a CL:PE ratio of 3:1 (75% CL) that corresponds to the conditions of bacterial stress. Our attention was still focused on the *a*-subunit to study the membrane lipid composition’s influence on the structure and hydration of the proton half-channels.

The described structure of proton half-channels was stable in all types of membranes. The proton transfer pathway presumably included a large number of water molecules and polar amino acid residues located inside the half-channels and were fully unreachable by the lipids. The obtained results confirm the generally accepted theory about the interaction of the polar head groups of cardiolipins with the positively charged or aromatic amino acid residues on the protein surface. The presence of stable binding sites on the outer surface of the *a*-subunit indicates a strong influence of electrostatic interactions on the membrane–protein dynamic structure during molecular dynamics simulation. However, CL-protein interactions were of a transient stochastic character. This allows us to conclude that, most likely, the nature of these interactions is collective and the activity of ATP synthase is modulated by the microviscosity of the lipid medium. On the other hand, having CL present at the inlet/outlet of the half-channels appears to be essential for enhancing the hydration of water cavities, ultimately leading to increased stability in proton transport. Nevertheless, a complete comprehension of how lipids and ATP synthase interact necessitates further investigation.

CL in the membrane obviously increased the hydration of the half-channels. The presence of CL binding sites near highly mobile loops of *a*-subunit also could lead to the penetration of more water molecules into the entrance lacuna, that led to the merging of two water cavities into one at the inlet half-channel entrance. The significant manifestation of this effect was also the emergence of a long continuous chain of water molecules to pass a proton directly to *c*Asp61 from the periplasm. In addition, the CL location near the inlet half-channel entrance could presumably contribute to the perimembrane localization of the protons, which in turn will lead to minimization of the pH changes in the periplasm.

Minor conformational changes in the half-channels that occurred with the addition of CL caused extremely rare direct transitions between *a*Glu219-*a*Asp119, *a*Glu219-*a*His245, and *a*Gln252-*c*Asp61. At the same time, deeper penetration of water molecules, in particular W2 and W3, also increased the stability of the proton transport continuity under conditions close to in vivo. The stable spatial positions of the significant amino acid residues *a*Asn214 found under all simulation conditions indicate a prevailing influence on the side-chain dynamics of local interactions between amino acids and water molecules. Increased hydration in the *a*Asn214 region (W2) that was also observed at 75% CL content, lead to the emergence of a new stable spatial position of SP2 (previously not observed) in *a*Asn214. Another example of a deeper penetration of water is the presence of several molecules near the key *c*Asp61 (corresponding W1 in the model POPC membrane). This significantly increases the probability of *c*Asp61 protonation, thereby ensuring the continuity of the protein functioning process.

The participation in proton transport of *a*Asn116 and *a*Ser144 residues within the *a*-subunit is under discussion as they are not included in the “classical” proton transfer pathway. The probability of their protonation directly or through water molecules (in the presence of CL) can be a factor regulating the rate of proton transport being a dead end or a trap. The outlet half-channel, which is a cavity filled with water, is a stable structure that weakly depends on the membrane’s composition. It is not known whether increasing the number of water molecules inside it has any effect on the proton transport, given the fact that they are in excess, possessing an average of more than 30 molecules in all simulations. An increase in the cardiolipin content in the membrane during adaptation to bacterial stress is also a subject of discussion, however, we did not find a major structural effects on the functioning of the F_o_ factor.

Thus, in our study, we confirmed the structural stability of F_o_F_1_-ATP synthase half-channels to changes in the membrane lipid composition. Mobility and the presence of stable spatial positions of significant amino acid side chain *a*Asn214 are important components that determine the characteristics of proton transport. Meanwhile, water molecules are obligatory participants in the proton transport chain through F_o_F_1_-ATP synthase, both as part of large water lacunae (according to the Grotthuss mechanism) and due to deep penetration into the protein (W1–W3), where their presence is a necessary condition for ensuring transport continuity. Increased hydration in the W1 region, in the presence of CL under conditions close to in vivo, enlarges the probability of the key *c*Asp61 protonation. At the same time, the formation of the continuous well-organized water molecules chain in in vivo membranes with CL can ensure the functioning of the enzyme by increasing the rate of proton movement along water molecules, as well as maintaining transport under conditions of critical mutations.

This is the first study that evaluated the effect of the membrane lipid composition on the proton half-channels’ structure and hydration in the *E. coli* ATP synthase, which are extremely important to take into account at simulation under conditions close to in vivo. Further research of the various factors’ influence on the process of proton transport will reveal the features of the cell energy supply of under physiological and pathological conditions.

## Data Availability

All material that could be shared is in this paper.
